# Femtosecond-laser-written Microstructured Waveguides in BK7 Glass

**DOI:** 10.1038/s41598-018-28631-3

**Published:** 2018-07-10

**Authors:** George Y. Chen, Fiorina Piantedosi, Dale Otten, Yvonne Qiongyue Kang, Wen Qi Zhang, Xiaohong Zhou, Tanya M. Monro, David G. Lancaster

**Affiliations:** 1 Laser Physics and Photonic Devices Laboratories, School of Engineering, University of South Australia, Mawson Lakes, South Australia 5095 Australia; 2 State Key Joint Laboratory of ESPC, School of Environment, Tsinghua University, Beijing, 10084 China

## Abstract

There is a deficiency of low-loss microstructured waveguides that can be fabricated with a single laser-pass to minimize stress build-up, which can enable enhanced functionality and higher compactness for integrated optical devices. We demonstrate, for the first time, a series of multi-ring claddings each with a pair of cores in BK7 glass. Each waveguide was fabricated using only a single laser-pass at 1 MHz pulse repetition rate, 5 mm/s translation speed, 250 fs pulse width, over a set of pulse energies. We obtained the lowest-reported propagation loss of 0.062 dB/cm, measured at 1155 nm wavelength from the waveguide written with 340 nJ pulse energy. The maximum observed numerical aperture is 0.020, measured at 1155 nm wavelength from the waveguide written with 620 nJ pulse energy. Such waveguides could be incorporated in integrated Raman laser platforms for biomedical applications.

## Introduction

Unlike many optical fiber technologies, integrated optical devices can be more readily miniaturized and unified with micro-electronics, realizing precise and highly complex integrated systems. Femtosecond-laser writing was first demonstrated in 1996^1–3^, and has since been extensively researched. Femtosecond-laser (fs) waveguide writing in glass is a promising fabrication technique for integrated optics, due to its versatility to rapidly direct-write complex structures with fine precision. In comparison, photolithography^4^ and focused ion beam micro-machining^5^ are slower. Waveguide structures can now be directly written in a multitude of materials, including glasses, crystals and polymers^6–8^. Compared to the most common techniques, electron beam lithography and plasma-enhanced chemical vapor deposition (PECVD), fs-laser direct writing has the benefits of fast fabrication, flexibility in waveguide design, high spatial precision (i.e. limited by beam quality, wavelength and polarization), and simple integration of the resulting waveguides with fiberized components^9^.

Of particular interest are borosilicate glasses, such as the borosilicate-crown glass from Schott (N-BK7, refractive index is 1.5055 at 1155 nm wavelength^10^), which exhibit high transmission in the visible and near-infrared, few defects, low density, high chemical-stability, and low processing-cost. Owing to these qualities, they are extensively used in commercial optics and in the optical communications industry, and waveguide writing in this material has attracted considerable interest.

The process of laser-induced waveguide fabrication can be categorized into three main types based on the formalism presented by Calmano *et al*.^11–13^. The Type I writing process (i.e. modifying the core region to change the core refractive-index) is simple, involving a single laser-pass (i.e. one-step motion of laser writing). Propagation losses as low as 0.2 dB/cm have been demonstrated via this type of writing^14^. However, multiple laser-passes are needed to fabricate a microstructured waveguide via a Type I writing process. The Type II writing process (i.e. modifying the cladding region to change both the core and cladding RIs) and the Type III writing process (i.e. modifying the cladding region to change the cladding RI alone) requires even more laser passes than the Type I writing process to create a microstructured waveguide, and engenders additional defects such as a highly scattering core-cladding interface.

It is difficult to fabricate low-loss microstructured waveguides in a BK7 glass substrate without multiple laser-passes, which increases production time and elevates the risk of stress-induced micro-cracks^15^ from successive heating and quenching cycles. However, such designs (e.g. two cores) supporting space-division multiplexing of light can offer enhanced functionality and higher compactness for integrated optical devices such as optofluidic chips^16^, photonic circuits^17^ and frequency combs^18^. Similar applications to those of twin-core fibers can also be explored, such as interferometric^19^-and plasmonic-based sensing^20^, and optical tweezers^21^. One study^22^ made progress towards fabricating microstructured designs with a single laser-pass, having written both a core and a single-ring cladding via a Type I writing process.

To address the problem of single laser-pass writing of microstructured waveguides, we have determined suitable parameters for writing to create waveguides comprising a multi-ring cladding and a pair of cores in a BK7 glass substrate, using the single-pass Type I writing process that would otherwise only be possible with a multiple laser-pass Type II writing process. Since only a single laser-pass is required per waveguide, it mitigates the build-up of stress inside the glass substrate, allowing further laser modifications in close proximity without cracking^15^. Applications employing differential techniques such as interferometry can benefit from the simplicity, configurability and robustness offered by this single-pass multi-core architecture.

## Fabrication

Nonlinear absorption of fs-laser pulse(s) followed by rapid ionization inside a bulk material leads to thermally induced formation of defects, material densification/rarefaction or material ablation. The introduction of these defects and density changes give rise to RI changes. The extent of such modification is determined by many factors such as wavelength, pulse energy, focal spot size, intensity/phase profiles, pulse width, pulse repetition rate, translation speed, writing direction, writing depth and polarization^2,3^. The parameter space for writing waveguides in glass is important to realizing the intended design and properties.

**Figure 1:**
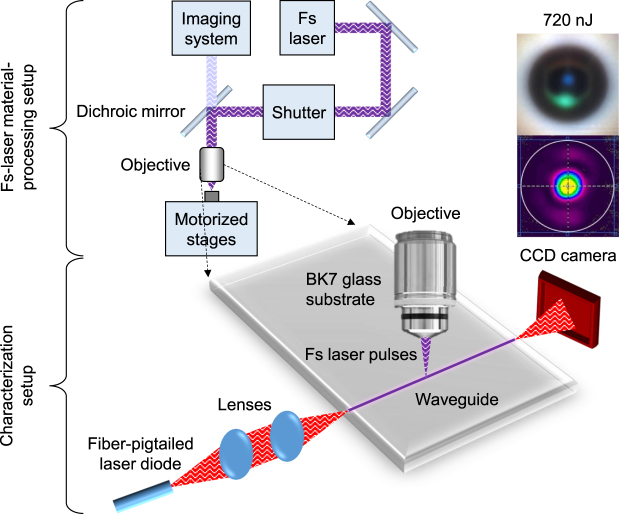
Schematic of the fs-laser material processing system, and the characterization method. Inset (top): cross-section image of waveguide written with 720 nJ pulse energy, inset (bottom): false-color intensity distribution of the centrally guided mode.

The femtosecond-laser material-processing system shown in Fig. 1 employed for this work was configured with 1047 nm wavelength, 250 fs pulse width, using 1.25 numerical aperture (NA) infinity-corrected microscope objective lens (Zeiss N-Achroplan) with 100 × magnification (i.e. to increase intensity and thus refractive-index change) and a working distance of 460 μm (i.e. refractive index of oil immersion matches that of BK7). Waveguide fabrication was performed using a pulse repetition rate of 1 MHz. Pulse energies were varied by using a variable attenuator (Altechna 2-UWPA-R1-0800). Laser passes were performed by translating the glass substrate mounted on a 4-axis air-bearing translation system with a positional accuracy of ~100 nm. To ensure sample flatness, a 2-axis goniometer is used to align the glass substrate face with the translation plane. To align the focal spot within the glass substrate, an imaging system is mounted above the objective lens. The imaging system consists of a CCD camera (Edmond Optics EO-5012C) fitted to a variable focal-length lens located ~250 mm from the back aperture of the objective lens. The dichroic mirror noted in Fig. 1 reflects a narrow wavelength band around the laser wavelength to the sample and allows transmission of the sample illumination to the imaging system. In this configuration, the imaging system and the objective lens forms a simple microscope with the laser beam included in the field of view of the microscope. To facilitate a stable laser beam to be focused down onto a sample, the system is built on and around a granite bridge platform resting on a passively vibration-isolated optical table.

A pre-objective spot diameter of ~5 mm, pulse width of 250 fs, pulse repetition rate of 1 MHz, linear polarization parallel to the written waveguides, laser translation speed of 5 mm/s (i.e. 200,000 pulses/mm and 0.2 s/mm), and writing direction from left to right were applied to write waveguides at 200 μm depth and 200 μm spacing in a BK7 glass substrate measuring 30.0 × 10.0 × 1.5 mm. Then, the end-faces of the waveguides were polished back by roughly 1 mm each to remove waveguide sections affected by boundary effects, resulting in the final dimensions of 30.0 × 8.1 × 1.5 mm.

## Results and Discussion

### Geometry measurement

**Figure 2:**
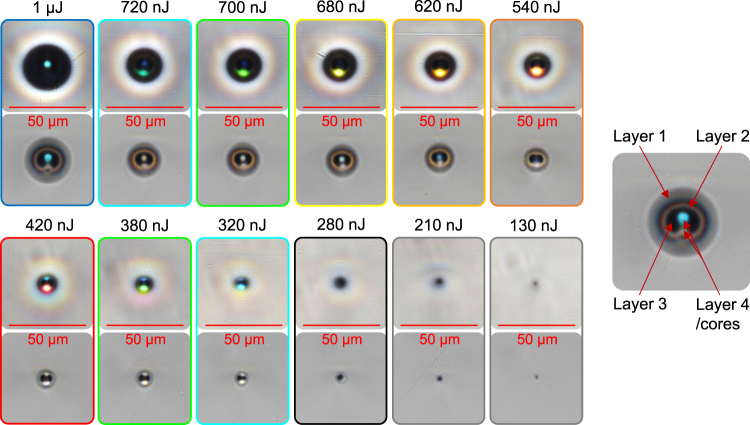
Bright-field transmission images of the waveguides written as a function of pulse energy. Top image of each pair: smaller illumination aperture, bottom image: larger illumination aperture.

To inspect waveguide geometry and light guidance, a halogen light bulb with an adjustable aperture was used in conjunction with a microscope (Nikon DS-Ri2) to observe the end-face of the waveguides. The resulting images are presented in Fig. 2 as a function of pulse energy, at both a smaller and larger illumination aperture sizes. The images are oriented such that the writing beam is incident from the top of each image. They exhibit varying sizes, structures and colors depending on the pulse energies used for writing, and different spectral transmission characteristics depending on the illumination aperture size.

A common feature of all waveguides is a non-circular geometry with one side of the multiple ring layers featuring a dip/spot (i.e. attributed to beam astigmatism, self-focusing and plasma defocusing^23^), featuring two cores aligned perpendicular to the glass interface seen from the longitudinal perspective. The multiple layers could be caused by laser-induced heating and quenching leading to material expansion and glass transitions. The dual-core formation could be due to the two focal-spots of the laser beam creating individual heating zones. However, as pulse energy is increased, the following trends were observed: (a) the waveguides are generally larger; (b) the evolution from one-layer to four-layer RI modifications consisting of lower, higher, lower, higher RI relative to the pristine bulk, judging by the different intensities of the guided light; (c) a change in guidance region geometry, from a pair of spots to a spot and a ring/spot; and (d) a change in wavelength selectivity in the form of color shift from blue, green, yellow, red and back again, obtained using a small illumination aperture.

**Figure 3:**
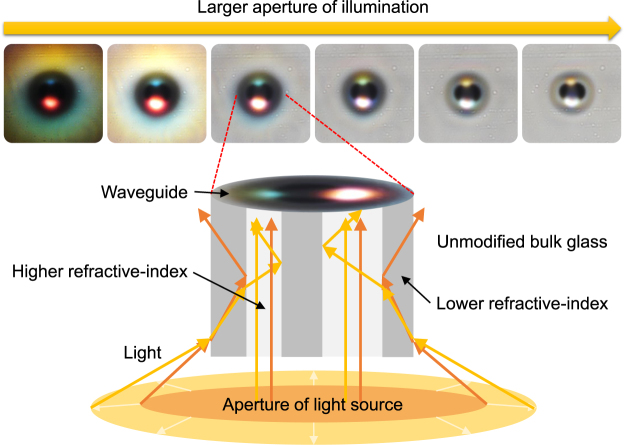
(top) Evolution of colors and structural clarity seen from the waveguide written with 420 nJ pulse energy, as a function of the size of the illumination aperture, with (bottom): illustration of wider apertures allowing more light to enter the waveguide, which experiences chromatic dispersion before a larger portion of light is guided.

The colors of the waveguides shown in Fig. 2 are attributed to wavelength-dependent scattering and chromatic dispersion of white light. The chromatic-dispersion contribution is illustrated in Fig. 3, where the diameter of the illumination aperture was varied for the waveguide written with 420 nJ pulse energy. With a small illumination aperture, the guided mode appears predominantly red followed by blue light. As the illumination aperture increased, the side-penetrated light experienced the following: (a) its incidence angle from the normal to the waveguide-bulk interface decreased; (b) a wavelength-dependent portion entered, became guided, accumulated, and then became the predominant wavelength. At larger illumination aperture sizes, the waveguide is over-filled (i.e. guides a larger volume of modes), and thus the waveguide structure is clearer. The bright ring surrounding the dark outer cladding of each waveguide is a common phenomenon, associated with stray light outside the waveguide being reflected by the waveguide cladding such that a relatively high portion of light is gathered around the outside of the waveguide.

The measurement of the refractive-index (RI) profile of the waveguides was attempted with an RI profiler (Rink Elecktronik). However, significant distortions and errors were observed due to complex refraction of light through the microstructured waveguide with the refracted near-field technique^24^.

### Numerical-aperture and loss measurement

**Figure 4:**
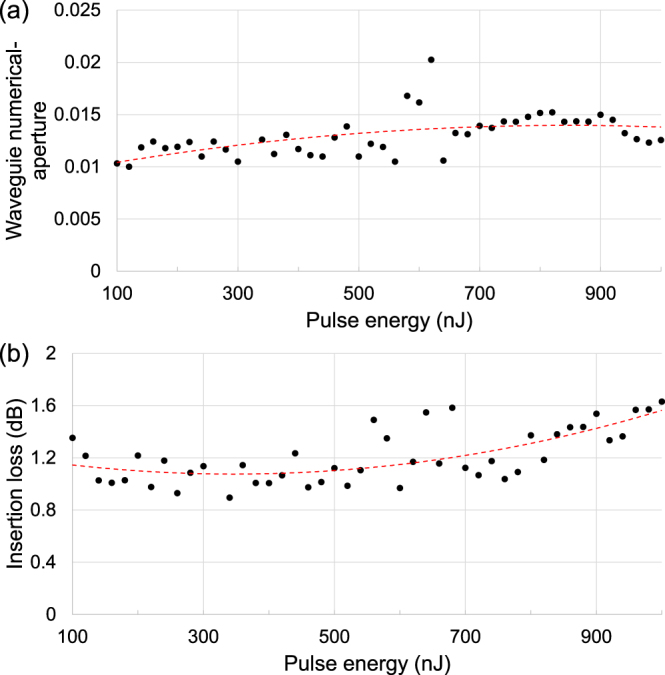
Relationship between pulse energy and (**a**) waveguide numerical-aperture; and (**b**) insertion loss. Curve loose-fitting with 2nd order polynomial for revealing general trends are represented by dashed lines. Waveguide lengths are 8.1 mm.

To measure optical losses and waveguide NA of the central core of each waveguide, polarized light from a laser diode of 1155 ± 5 nm wavelength with a polarization-maintaining fiber pigtail was launched into a waveguide using a 60 mm focal-length plano-convex lens, as shown in Fig. 1. The output far-field intensity profile was observed with a pyroelectric array camera (Ophir Pyrocam IV). A software-based virtual circular-aperture was used to only consider light from the waveguide, by placing it within the far-field ring-shaped gap separating the circular beam from the background light. The ring-shaped gap exists due to a combination of Fresnel filtering^25^ and background light reflecting off the waveguide cladding prior reaching the output end where the guided light begins to diverge. The loss measurements were performed with a linear polarization aligned parallel to the substrate faces (i.e. horizontal to waveguide images shown in Fig. 2). The maximum observed NA measured at 1155 nm wavelength is associated with the waveguide written with 620 nJ pulse energy. At a distance of 175.7 mm between the waveguide output end-face and the pyroelectric camera sensor, and a far-field intensity radius of 3.557 mm (D4σ), the divergence angle was determined to be 0.020 rad, and thus waveguide NA based on the sine of the divergence angle is 0.020. As an estimate, the RI change of the core is. Figure 4(a) shows a trend between pulse energy and waveguide NA, where the peak of the curve loose-fitting occurs around 800 nJ. The actual peak occurs at a lower pulse-energy of 620 nJ, possibly due to an optimum combination of RI modifications between different layers of the microstructured waveguide. The waveguide-NA errors due to system noise, image-processing uncertainties and measurement repeatability errors are too small to be visible, and thus not plotted.

**Figure 5:**
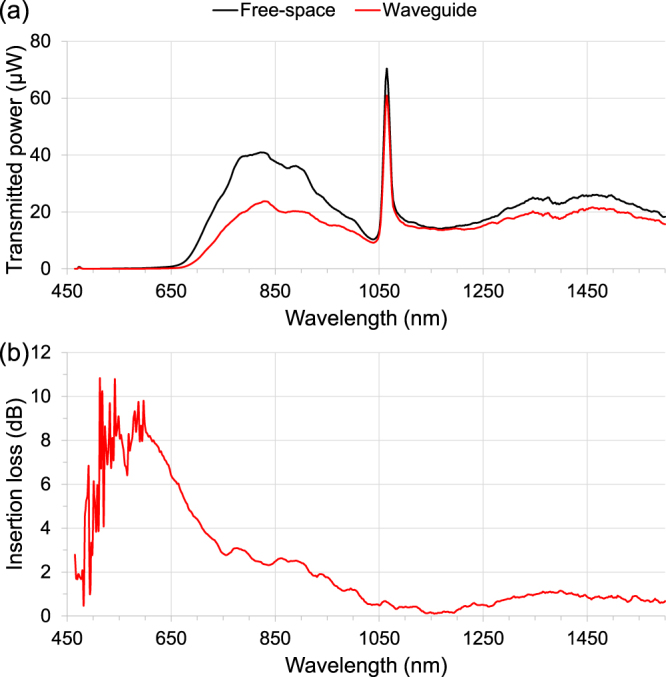
(**a**) Transmittance; and (**b**) insertion loss; of the waveguide written with 340 nJ pulse energy. Waveguide length is 8.1 mm.

To gain an insight into the wavelength dependency of insertion loss, broadband light from a supercontinuum laser source (YSL SC5, 470–2400 nm) was launched into each waveguide. The output light was collimated then focused into a single-mode optical fiber connected to an optical spectrum analyzer (Ando AQ-6315E). Note it is not critical that all the light is captured by the optical spectrum analyzer, as the shape of the insertion-loss spectrum is the primary interest. In addition, the alignment was optimized for maximizing the transmitted power of the central region of the spectral range, and any adjustments resulted in changes in transmitted power mainly at the two ends of the spectrum. Therefore, the transmitted power below 650 nm and above 1560 nm were not accurate measured. The insertion loss (positive number format) of a waveguide is straightforward to measure, being 10 times the logarithmic ratio between the waveguide transmitted power and the free-space transmitted power. Figure 5(a) plots the transmission spectrum of the supercontinuum laser source through free-space (i.e. light focused through air instead of substrate) compared to that of the waveguide written with 340 nJ pulse energy. The peak at 1064 nm indicates the residual pump light. Figure 5(b) reveals the corresponding insertion losses, where low-loss regions can be found at ~475 nm and ~1155 nm. It was found that a number of waveguides exhibit low insertion-loss between 1150–1200 nm. This spectral range is useful for propagating light emitted from integrated Raman lasers^26^ for biomedical applications. The insertion losses measured below 650 nm are unreliable, due to the low signal-to-noise ratios.

The coupling loss and the propagation loss were derived by solving simultaneous equations, Equation 1 and Equation 2, involving two waveguides written with the same parameters except different lengths. The insertion losses were measured with the CCD camera using the aforementioned method. The Fresnel reflection loss was calculated with knowledge of the RI of BK7 glass (1.5055 at 1155 nm wavelength). The waveguide lengths were measured with a Vernier calliper.

1 $${a}_{1}+{a}_{2}{L}_{1}+2{a}_{3}={a}_{4}$$

2 $${a}_{1}+{a}_{2}{L}_{2}+2{a}_{3}={a}_{5}$$

where *a*_1_ is the coupling loss (dB), *a*_2_ is the propagation loss (dB/cm), *L*_1_ is waveguide length #1 (cm), *L*_2_ is waveguide length #2 (cm), *a*_3_ is the Fresnel reflection loss (dB), *a*_4_ is insertion loss #1 (dB), and *a*_5_ is insertion loss #2 (dB). The values of *a*_1_ and *a*_2_ can be deduced as follows:

3 $${a}_{2}=({a}_{5}-{a}_{4})/({L}_{2}-{L}_{1})$$

4 $${a}_{1}={a}_{4}-{a}_{2}{L}_{1}-2{a}_{3}$$

The lowest-loss waveguide measured at 1155 nm wavelength was written with 340 nJ pulse energy, with *L*_1_ = 8.1 mm, *L*_2_ = 34.2 mm, *a*_3_ = 0.180 dB, *a*_4_ = 0.725 dB, *a*_5_ = 0.888 dB, which yields *a*_2_ = 0.062 dB/cm, and *a*_1_ = 0.315 dB. We speculate that that the interplay of 1 MHz pulse repetition rate, 1047 nm wavelength and 250 fs pulse width gave rise to suitable intensity and heating/quenching conditions for low-loss waveguide formation. The polarization-dependent loss is 0.4 dB (i.e. length-independent), measured by inserting a half-wave plate (i.e. mechanical rotation induces rotation of the polarization state) followed by a polarization beamsplitter cube (i.e. measures peak-to-peak difference in transmitted power with respect to all possible states of polarization) between the lens and the optical fiber. The lowest propagation-loss reported is associated with the aforementioned state of polarization, where the linear polarization was aligned parallel to the substrate faces. The circular symmetry of the guided mode is lower for the orthogonal state of polarization. Figure 4(b) reveals a trend between pulse energy and insertion loss, where the valley of the curve fitting occurs around 340 nJ. Waveguides fabricated from a writing direction of right-to-left laser translation result in much higher insertion-losses. The fluctuation of the insertion loss for different writing pulse-energies is not attributed to system noise nor measurement errors, because repeating the waveguide loss measurement yields the same results. The insertion-loss errors due to system noise, image-processing uncertainties and measurement repeatability errors are too small to be visible, and thus not plotted. The central core generally exhibited slightly better quality of mode guidance in terms of a near-Gaussian intensity profile. The waveguide loss and NA between the two cores are similar.

**Figure 6:**
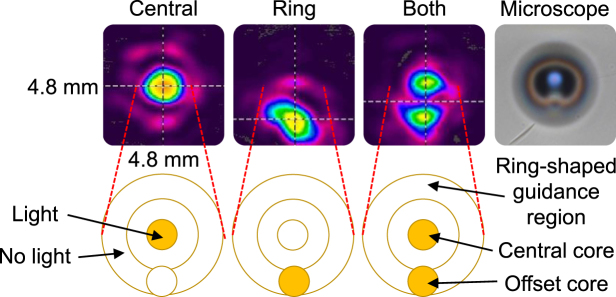
Comparison of the false-color far-field intensity profile of different guiding configurations for the waveguide written with 900 nJ pulse energy. Inset: illustration of the excited waveguide regions.

To show that light can be launched into either the central core and/or the offset core adjacent to the ring-shaped guiding region, the 1155 nm laser diode was used in conjunction with lenses to probe the waveguide written with 900 nJ pulse energy. Unfortunately, the ring-shaped guiding region is slightly elliptical. If it could be improved, possibly via the beam-reshaping slit method^27^, it might be useful for optical manipulation via vortex modes^28^. Figure 6 shows that the mode excitation can be tailored by defocusing and changing the radial offset of the input light. A weak evanescent cross-talk between the cores was observed, judging by the faint overlap between the guidance regions. It is unlikely to be a higher mode excitation, judging by the separation distance between the two spots when viewed close to the waveguide output end-face. Nevertheless, all the guiding regions are not strictly single-mode, while the central core approaches single mode guidance, judging by its circular symmetry.

## Conclusion

We have demonstrated a single-pass fs-laser writing technique to fabricate up to an unprecedented 4 layers of refractive-index modification, resulting in multi-ring waveguides and with pairs of cores. A single pass is advantageous, because it reduces fabrication time and lowers the build-up of stress inside the glass substrate. Two cores and a guiding ring enhance the functionality of the resulting device. This single-pass multi-core architecture delivers simplicity, configurability and robustness, which is the key for fabricating the building blocks of complex yet compact integrated-optic circuits and sensors. A simple application demonstration was also given in the form of an interferometric temperature sensor.

The determined suitable parameters for writing consist of 1 MHz pulse repetition rate, 5 mm/s translation speed, 250 fs pulse width, and a range of pulse energies. We obtained the lowest-reported propagation loss of 0.062 dB/cm, measured at 1155 nm wavelength from the waveguide written with 324 nJ pulse energy. The maximum observed numerical aperture is 0.020, measured at 1155 nm wavelength from the waveguide written with 620 nJ pulse energy.

## Experimental Section

### Preparation for waveguide writing

Prior to irradiating the BK7 glass substrate with fs-laser pulses, the substrate was cleaned using a lens wipe pre-wetted with isopropanol. Then, the substrate position and laser beam were aligned with the translation stages by using cameras and the mirror/aperture method respectively. After that, immersion oil (Zeiss Immersol 518 N) was uniformly deposited on the substrate surface. This provides a means to align the tilt angles of the substrate relative to the laser beam as well as to find the depth of the focal point of the laser beam, by producing observable backscattered-light when the laser is focused in the oil layer.
